# Interaction of Multimicrobial Synthetic Inhibitor 1,2-Bis(2-Benzimidazolyl)-1,2-Ethanediol with Serum Albumin: Spectroscopic and Computational Studies

**DOI:** 10.1371/journal.pone.0053499

**Published:** 2013-01-04

**Authors:** Nayana Kamtekar, Anita Pandey, Neeraj Agrawal, Raghuvir R. S. Pissurlenkar, Mohanish Borana, Basir Ahmad

**Affiliations:** 1 UM-DAE Centre for Excellence in Basic Sciences, University of Mumbai, Kalina Campus, Mumbai, India; 2 Molecular Simulations, Pharmaceutical Chemistry Division, Bombay College of Pharmacy, Mumbai, India; Russian Academy of Sciences, Institute for Biological Instrumentation, Russian Federation

## Abstract

The molecule, 1,2-Bis(2-benzimidazolyl)-1,2-ethanediol (BBE) is known to act as a selective inhibitor of poliovirus, rhinovirus, *Candida albicans*, several bacterial species, and is easily synthesized by Phillips reaction. The interaction of BBE with BSA and the effects of its binding on the conformation and unfolding/refolding pathways of the protein were investigated using multispectroscopic techniques and molecular modeling. The binding studies indicate that BSA has one high affinity BBE binding site with association constant 6.02±0.05×10^4 ^M^−1^ at 298 K. By measuring binding at different temperatures, we determined the changes in enthalpy (ΔH = −15.13±2.15 kJ mol^−1^), entropy (ΔS = 40.87±7.25 J mol^−1^ K^−1^) and free energy (ΔG_ = _26.78±1.02) of interaction, which indicate that the binding was spontaneous and both enthalpically and entropically driven. Based on molecular modeling and thermodynamic parameters, we proposed that the complex formation involved mainly hydrophilic interaction such as hydrogen bonding between hydroxyl groups of ethane-1,2-diol fragment with Tyr410 and benzimidazole sp^2^ nitrogen atom with Ser488 and hydrophobic interaction between phenyl ring of one benzimidazole of the ligand and hydrophobic residues namely, Ile387, Cys391, Phe402, Val432 and Cys437. The sequential unfolding mechanism of BSA, site-specific marker displacement experiments and molecular modeling showed that the molecule preferably binds in subdomain IIIA. The BBE binding to BSA was found to cause both secondary and tertiary structural alterations in the protein as studied by intrinsic fluorescence, near-UV and far-UV circular dichroism results. The unfolding/refolding study showed that BBE stabilized native to intermediate states (N
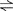
I) transition of the protein by ∼2 kJ mol^−1^ without affecting the intermediate to unfolded states (I
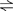
U) transition and general mechanism of unfolding of BSA.

## Introduction

Synthetic chemistry and ligand-protein interaction studies have played an important role in speeding up the recent drug discovery processes. The methods of synthetic chemistry create small molecules rapidly for screening, and ligand-protein interaction studies provide information on how a potential drug interacts with target protein [1], [2]. The knowledge of ligand-protein interaction is also vital for the accurate prediction of its biological function [3]. Therefore, the characterization of a synthetic potential drug molecule interaction with proteins is mandatory and requires determination of association constant, number of binding sites, thermodynamic properties, binding induced conformational alterations and stability of the protein. It is now understood that the ligand residence time, the total duration of ligand-protein binding have a vital impact on the ligand’s effectiveness and selectivity [4], [5].

**Figure 1:**
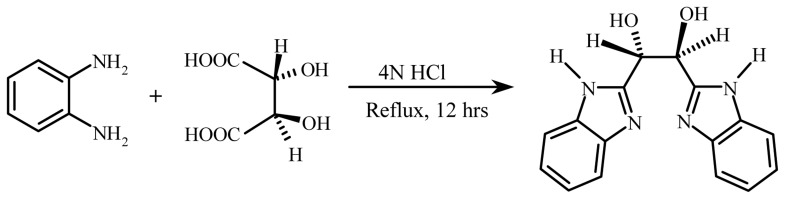
Preparation and structure of 1,2-Bis(2-Benzimidazolyl)-1,2-Ethanediol (BBE).

Several studies have reported that 1,2-Bis(2-benzimidazolyl)-1,2-ethanediol (BBE) acts as a selective inhibitor of poliovirus [6], [7] and rhinovirus infections [8]. The compound and its metal ion complexes also show broad-spectrum antibacterial activity against *Staphylococcus aureus, S. epidermis, Klebsiella pneumoniae, Salmonella typhi* and antifungal activity against *Candida albicans* [9]. The molecule 1,2-Bis(2-benzimidazolyl)-1,2-ethanediol (BBE) is a chiral tridentate ligand. It can be prepared with Tartaric acid and 1,2-diaminobenzene by a simple Phillips reaction [10] (Figure 1). Despite broad inhibitory effects against all kinds of pathogens and the ease of synthesis, its interaction with target pathogen proteins or drug transport proteins, such as serum albumin, is not known.

With this perspective, we carried out detailed investigations of the interaction of BBE with bovine serum albumin (BSA), which shares ∼80% sequence homology with human serum albumin and contains almost identical ligand binding sites for drugs and other endo- and exogenous ligands [11]. Previous studies on ligand-serum albumin binding showed that subdomain IIA and IIIA of the proteins contain the main binding sites for drugs [12], [13]. The BBE interaction to serum albumin cannot be expected to explain the effect of the molecule directly on target pathogen protein but it may be used as a model to study the effect of the molecule on pathogen proteins. Moreover, serum albumin is the major transport and depot protein of mammalian systems. The bioavailability, distribution and metabolism of a drug are largely controlled by its interaction with serum albumin [14], [15].

In this study, we investigate the BBE-BSA interaction mechanism and characterize the binding parameters (K_a_ and n), binding location, thermodynamics parameters (ΔH, ΔS and ΔG) and residence lifetime (τ) of the complex. We also show the effect of interaction on conformation, unfolding/refolding pathways and stability of the protein. We used the widely applied fluorescence quench titration method to investigate the binding mechanism [16], [17]. The molecule binding location was determined by using methods such as sequential unfolding mechanism of serum albumin [18], displacement of site-specific marker [19] and molecular modeling. The conformational alteration and unfolding/refolding studies were made by using far-UV CD as a probe for secondary structure and intrinsic fluorescence and near-UV CD as probes for tertiary structure.

## Materials and Methods

### Materials

All Chemicals and reagents were purchased from Sigma-Aldrich or SD fine chemical Ltd. The ligand 1,2-bis(2-benzimidazolyl)-1,2-ethanediol (BBE) was synthesized according to the reported procedure [10] and purity was checked by thin layer chromatography, melting point and ^1^H NMR. Spectroscopic studies were performed using spectrophotometer (Shimadzu UV 1800), spectrofluorimeter (Fluoromax 4, Horiba), and Circular dichromism (Jasco J-815).

### Interaction Studies of BBE with BSA

Bovine serum albumin stock solution (150 µM) was prepared in 60 mM sodium phosphate buffer of pH 7.4 and the concentration was determined spectrophotometrically using 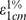 of 6.67 at 279 nm [20]. BBE stock solution (2.7 mM) was prepared by dissolving 1 mg of the compound in 1 mL methanol. For the binding studies, to a fixed volume (3.0 mL) of 5 µM protein solution was added an increasing volume (0−20 µL) of the ligand. The fluorescence was measured between 300 and 400 nm after exciting the samples at 280 nm. The effect of highest concentration of methanol (0.66% v/v) added in the samples during titration was investigated on the far-UV CD spectrum and intrinsic fluorescence spectrum of the protein. We found that both far-UV CD and intrinsic fluorescence spectra of BSA were almost identical in the absence and presence of 0.66% v/v methanol, which indicated that the conformation of BSA did not alter in the presence of highest concentration of methanol (0.66% v/v) used in this study.

Site-specific marker displacement experiments were performed by titrating diazepam-BSA complex obtained by mixing the two at different diazepam/BSA molar ratios (0∶1, 0.5∶1, 1∶1, 2∶1) with increasing concentrations of BBE. The intrinsic fluorescence of BSA was recorded between 300–400 nm after excitation at 280 nm. Each spectrum was background subtracted with the spectrum of free BBE.

### Conformational Studies

#### Intrinsic fluorescence

Intrinsic fluorescence of BSA in the absence and presence of different concentrations of BBE was measured between 300 and 400 nm by exciting at 280 nm. The excitation and emission slits were set at 4 nm and the fluorescence spectra were taken at three different temperatures. Rayleigh scattering measurements in the absence presence of 0.01% Triton X-100 were performed at room temperature in a 1 cm path-length cell. Samples were excited at 350 nm and spectra were recorded in the range of 300–400 nm. Both excitation and emission slits were fixed at 5 nm. Data were plotted at 350 nm.

#### Far- and near-UV circular dichroism

Circular dichroism studies were carried out with a Jasco J-815 spectropolarimeter after calibrating it with D-10-camphorsulfonic acid under a constant nitrogen flow. Far- and near-UV CD spectra in the absence and presence of different molar ratios of BBE and BSA were taken at the protein concentrations of 5 and 25 µM with 0.1- and 1.0 cm path length cells, respectively. Each spectrum was the average of four scans and background subtracted with the spectrum of free BBE. The results were presented as mean residue ellipticity [θ] in deg cm^2^dmol^−1^, which is defined as
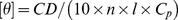
(1)where CD is in milli-degree, n is the number of amino acid residues (583), l is the path length of the cell in cm, and C_p_ is molar concentration of the protein.

The amount of regular secondary structures (α-helix and β-strand) were determined by analysis of the CD spectra using K2D3 deconvolution software (21).

### Protein Unfolding and Stability Studies

The unfolding/refolding studies of BSA upon BBE binding were carried out by urea denaturation of the protein. To a 100 µL stock BSA solution at pH 7.4 were first added different volumes of the 60 mM sodium phosphate buffer of pH 7.4, followed by the addition of stock urea (10 M) and BBE to get a desired concentration of denaturant and the ligand. The final solution mixture (3.0 mL) was incubated for 8–10 hours at room temperature and the unfolding process was monitored by intrinsic fluorescence at 343 nm by exciting the protein at 280 nm.

#### Unfolding data analysis

The unfolding transitions of BSA and ligand-protein complex followed a two-step three state mechanism and can be represented by the equilibrium equation
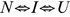
(2)where N, I, U are native, intermediate and unfolded states of the protein, respectively. In such type of transition, each step can be assumed to follow a two state mechanism [22]. The data monitored by intrinsic fluorescence at 343 nm were expressed in term of the fraction of intermediate state (*f_I_*) for the N
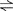
I transition and the fraction of unfolded state (*f_U_*) for the I
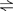
U transition calculated from following equations



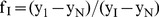
(3)

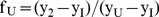
(4)


where *y_1_* and *y_2_* are the observed fluorescence intensity corresponding to first and second transitions, respectively. *y_N_*, *y_I_* and *y_U_* are the fluorescence intensities of the N, I, and U states of BSA and were obtained by linear regression of the data corresponding to pre-, intermediate and post-transition regions of unfolding profile, respectively. The changes in free energy of the two transitions at different urea concentration were calculated by following equations.



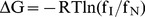
(5)

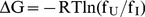
(6)


The free energy change in the absence of urea ΔG^H^_2_^O^ was determined by least square analysis of ΔG versus [urea] plot using equation



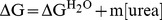
(7)The ΔG values were also determined by nonlinear regression of the data using equation.
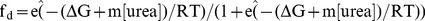
(8)where *f_d_* is the fraction unfolded (*f*_I_ and *f_U_*), R and T are universal *gas constant* (8.3145 J/mol) and absolute temperature (298 K), respectively. m is the measure of dependence of ΔG on urea concentrations.

### Computational Studies

The docking studies were carried out with the docking program Glide v5.8 [23], [24] module of Schrödinger Suite 2012 (Schrödinger LLC, USA) running on an Intel Xeon processor based HPC Cluster with Rocks 5.4 Cluster Suite as the operating environment.

### Protein and Ligand Preparation for Computational Study

The X-ray crystal structures of human serum albumin (PDB code 2BXF) and bovine serum albumin (PDB code 3V03) were retrieved from the Protein Data Bank [25], [26]. Due to the absence of bound ligand with BSA, the binding site was defined based on the facts that (i) BSA shares high sequence homology with HSA, and (ii) that BBE was found to interact with the diazepam binding site (subdomain IIIA), identified from the experimental studies. The coordinates of diazepam were extracted into the coordinate frame of BSA after alignment with the HSA, which is bound to diazepam in the subdomain IIIA.

The structure 3V03 was prepared for the docking studies with the Protein Preparation Wizard (Schrödinger Suite 2012). The protein BSA was structurally aligned to HSA after which the ligand (diazepam) coordinates were extracted in the BSA coordinate frame. The crystal waters were removed, hydrogen atoms were added, and atom types and partial charges were assigned based on the OPLS2005 force field. The formal charges for the acidic and basic amino acids were set according to the physiological pH 7.4. The missing side chains were added using the Prime v3.1 (Schrödinger Suite 2012). The complex system was relaxed using energy minimization protocol until an energy gradient of 0.01 kcal/mol/Å (1 cal = 4.184 J) was reached with the OPLS2005 force field.

The structure of 1,2-Bis(2-benzimidazolyl)-1,2-ethanediol was prepared using LigPrep v2.6 (Schrödinger 2012 Suite). The atom types and partial charges were assigned based on the OPLS2005 force field, corresponding to the physiological pH 7.4.

#### Docking studies

A grid around the bound ligand (diazepam) defined the active site of BSA. The docking grid comprised of two grid boxes - the inner grid box set to dimensions 10 Å^3^ while outer grid set to 20 Å^3^, thus providing ample space for the generation of diverse ligand conformations in the subdomain IIIA binding site by the search and score algorithm. The vdW radius scaling of 1.0 Å was applied to soften the potential in the nonpolar areas of the protein present within the grid extents with the partial atomic charges set to 0.25, while no scaling was applied to receptor atoms beyond the extent of the grid. The rotation of side-chain hydroxyl functions was allowed for certain amino acids namely serine, threonine, and tyrosine to increase the chances for the formation of hydrogen bonds with the polar ligand substitution.

The settings for the docking study were validated based on the protocol’s ability to reproduce the X-ray conformation of the bound ligand in the subdomain IIIA of HSA and subsequently of BSA.

The validated protocol was used to identify the docking solutions to the binding of BBE in the subdomain IIIA of BSA. The poses were ranked with scoring function GlideScore XP [27].

## Results

### 1,2-Bis(2-benzimidazolyl) 1,2-ethanediol (BBE) Induced Quenching Mechanism of BSA Fluorescence

**Figure 2:**
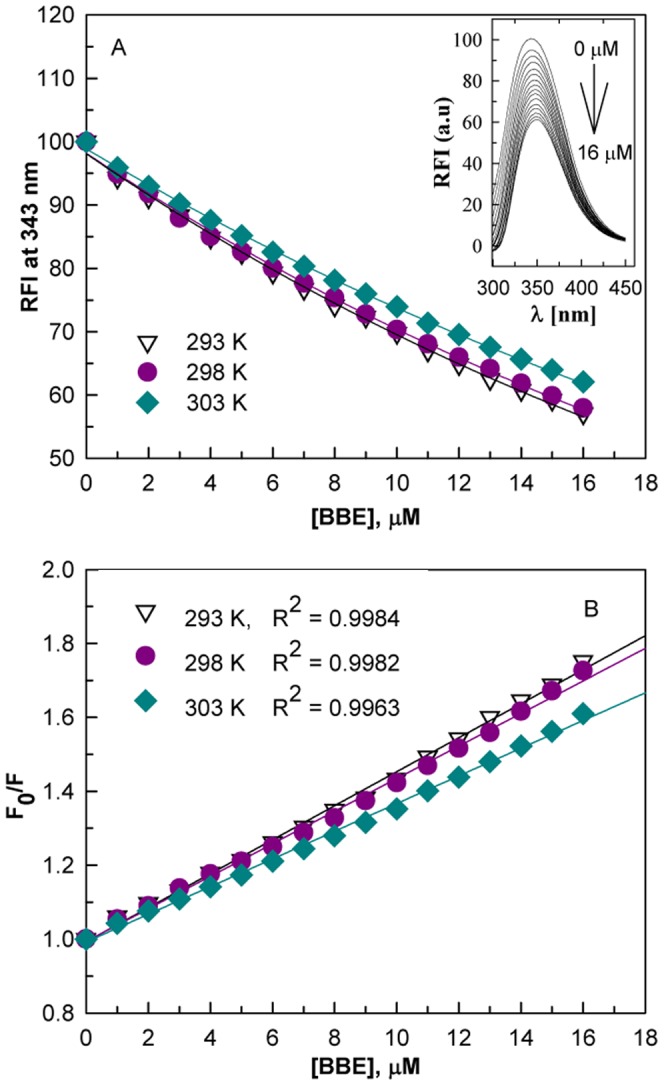
1,2-Bis(2-Benzimidazolyl)-1,2-Ethanediol (BBE) induced fluorescence quenching mechanism of bovine serum albumin. The BBE induced intrinsic fluorescence quenching (A) and Stern-Volmer plots for fluorescence quenching data (B) of BSA at different temperatures. The inset of Figure A shows fluorescence spectra of BSA in the absence and presence of increasing BBE concentrations at 298 K. The concentration of BSA was 5 µM and the intrinsic fluorescence of the protein was measured in 60 mM sodium phosphate buffer of pH 7.4 upon excitation at 280 nm.

The fluorescence quenching titration method has been extensively used to elucidate the ligand binding behavior of proteins [16], [18], [28]. Figure 2A shows the 1,2-bis(2-benzimidazolyl)-1,2-ethanediol (BBE) induced quenching of bovine serum albumin (BSA) fluorescence at different temperatures. The inset of Figure 2A shows the effect of different concentrations of BBE on fluorescence spectra of BSA at 298 K. It is apparent from the Figure 2 that equilibration of the protein with BBE caused concentration dependent quenching of the intensity and a gradual red shift (∼6 nm) in emission maximum (λ_max_) of BSA. These changes in the fluorescence properties of BSA suggest formation of stable complex with BBE.

To confirm the quenching mechanism, the data at different temperatures were analyzed according to the Stern-Volmer equation [29].
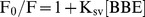
(9)where F_0_ and F are the fluorescence intensity in the absence and presence of the quencher (BBE) and K_sv_ is the Stern-Volmer constant. The K_sv_ at different temperatures were determined by linear regression of plots of F_0_/F versus [BBE] (Figure 2B). The K_sv_ for BBE at different temperatures were found to be of the order of 4×10^4^ M^−1^ (Table 1). Linear Stern-Volmer plots (regression coefficient R^2^>0.996) at all temperatures suggest that only one kind of quenching mechanism, static or dynamic, dominates. It also indicates that there is only one set of equivalent BBE binding sites on BSA. The Stern-Volmer quenching constant is given by k_q_τ_0,_ where k_q_ and τ_0_ are the bimolecular quenching rate constant and lifetime of the protein fluorescence in the absence of ligand, respectively. The lifetime τ_0_ of BSA has been accurately estimated to be 5×10^−9^s [30] and using the K_sv_ values in Table 1, k_q_ values at different temperatures were calculated and were found to be in the range of 10^13^ M^−1^s^−1^ (Table 1). These values were markedly larger than the maximum collisional quenching constant (10^10^ M^−1^s^−1^) [31], which indicated that BBE-induced quenching was due to complex formation. Moreover, a decrease in K_sv_ was measured with increasing temperature (Table 1), which also indicated that the BBE-induced quenching mechanism was due to complex formation. Taken together, the linearity of Stern-Volmer plots, decrease in Stern-Volmer constants and very high values of the quenching rate constants which increase with temperature support the fact that the BBE-induced quenching of BSA fluorescence is due to complex formation and BSA contains a single type of equivalent binding sites for the molecule.

**Table 1:** 1,2*-*Bis(2-Benzimidazolyl) 1,2-Ethanediol interaction and thermodynamic parameters.

T (K)	K_sv_ ×10^4^(M^−1^)	k_q_ ×10^12^ (M^−1^s^−1^)	K_a_ ×10^4^ (M^−1^)	n	−ΔH (kJ mol^−1^)	ΔS (J mol^−1^ K^−1^)	−ΔG (kJ mol^−1^)
293	4.59±0.06	9.18±0.12	6.85±0.07	1.04±0.03			27.10±1.06
298	4.40±0.07	8.80±0.14	6.02±0.05	1.03±0.02	15.13±2.15	40.87±7.25	26.78±1.02
303	3.74±0.04	7.48±0.08	5.58±0.05	1.04±0.04			26.06±0.97

**Figure 3:**
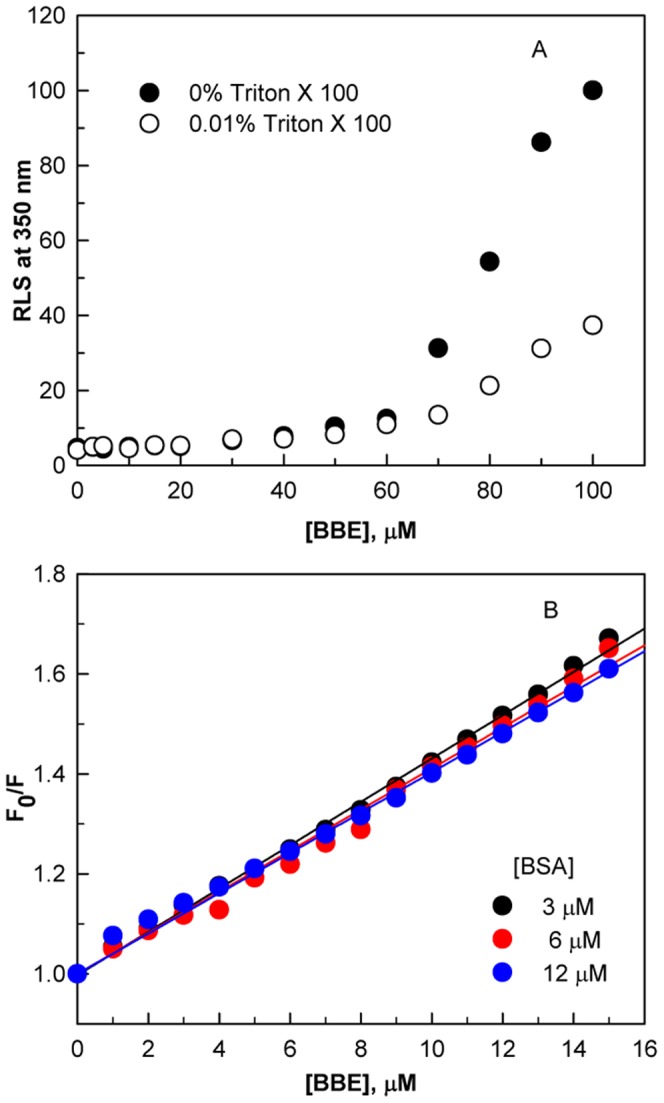
The BBE binding to BSA is specific. Particle formation by BBE in the absence and presence of 0.01% Triton X-100 as monitored by Rayleigh light scattering at 350 nm (A). Stern-Volmer plots of BBE-induced fluorescence quenching of BSA with increasing concentrations of the protein (B).

Many small molecules are known to form colloidal aggregates in aqueous solution at micromolar concentrations and adsorb proteins, which leads to artifacts in screens for ligands of proteins [32], [33]. To check that BBE is binding specifically to BSA, we monitored the colloid aggregate formation by BBE using Rayleigh scattering measurements at 350 nm and measurements of binding affinity at different BSA concentration. As can be seen from the Figure 3A, particles formation is not noticed upto 60 µM BBE concentration. Moreover, BBE binding profile does not alter significantly with increasing protein concentration (Figure 3B). The Ksv values are found to be 4.59×10^4^, 4.11×10^4^ and 4.03×10^4^ M^−1^ at 3, 6 and 12 µM BSA, respectively. Since it is know that colloid based sequestration of proteins significantly alter the binding affinity at higher protein concentration [33],we conclude that the BBE binds to BSA specifically.

### Interaction, Thermodynamic Parameters and Residence Time of BBE-BSA Complex

The BBE-BSA complex interaction parameters, association constant (K_a_) and number of binding sites (n) were determined by the following equation [34]

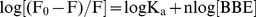
(10)


**Figure 4:**
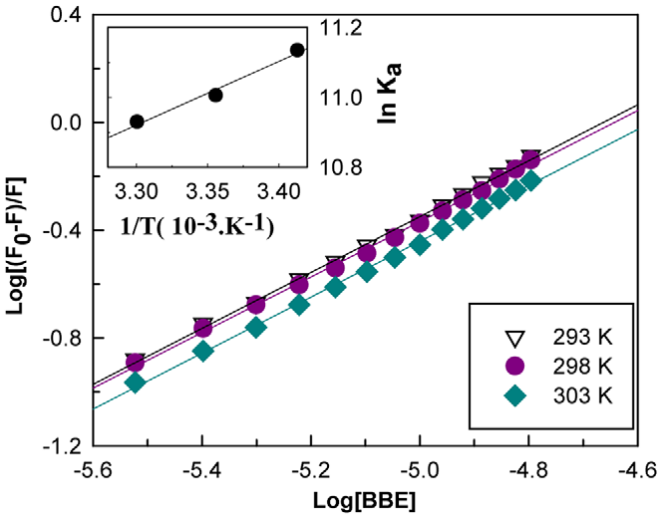
The binding and thermodynamic parameters of BBE-BSA complex. Log[{(F0-F)}/F ] versus log[Q] plots of the BBE-BSA binding data at three different temperatures for the determination of the association constant (K_a_) and number of binding sites (n). The inset shows the van’t Hoff plot for BBE-BSA interaction for the determination of thermodynamic parameters.

The least-square analysis of log[(F_0_-F)/F] versus log[BBE] plots gave straight lines at all temperatures whose slope were equal to n and intercept to logK_a_ (Figure 4). As shown in Table 1, BBE binds strongly to BSA with an association constant (K_a_) of ∼6×10^4 ^M and binding affinity (n) of ∼1. At the physiological temperature range (25 to 37°C), several exogenous and endogenous ligands bind to serum albumin with association constants between 10^3^ and 10^5 ^M [16], [34], [35], which are similar to the values of K_a_ and n obtained for our system (Table 1).

Using association constants (K_a_) at various temperatures (Table 1), we determined the change in enthalpy (ΔH) and entropy (ΔS) of interaction using the van't Hoff equation [36].
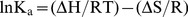
(11)where T is the temperature in Kelvin, and R is the gas constant (8.3145 J mol^−1^ ). The ΔH and ΔS were calculated from the slope and intercept, respectively, of the van’t Hoff plot between lnK_a_ and 1/T (inset Figure 4). Subsequently, the free energy changes (ΔG) of the reactions at different temperatures were calculated by substituting the values of ΔH and ΔS thus obtained using the equation, 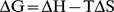. The values of thermodynamic parameters, ΔH, ΔS and ΔG are shown in Table 2. The high negative ΔH and positive ΔS values makes the ΔG value more negative for the BBE-BSA complex formation. This indicates that BBE-BSA complex formation is a spontaneous process and both enthalpically and entropically driven. These results suggest that binding forces involved are both hydrophilic interaction (hydrogen bonding) and hydrophobic interaction.

**Table 2:** 1,2*-*Bis(2-Benzimidazolyl) 1,2-Ethanediol induced conformational alteration in BSA.

BBE/BSAMolar ratio	K2D3% α-helices	K2D3% β-strand	[θ]_268_/[θ]_262_ ratio	λ_max_ Emission
0∶1	65.61±4.11	7.44±1.31	0.93	343.0
1∶1	63.08±3.27	7.81±1.44	1.16	345.5
2∶1	60.34±2.78	8,01±1.13	1.31	348.5

Currently, the residence time (τ) of protein-drug complex is considered as an important touchstone for both the dose response relationship and the rate of drug elimination [37], [38]. The residence time is defined as the reciprocal of the dissociation rate constant (τ = 1/k_b_). The residence time of BBE-BSA complex was determined with the kinetic model [16].
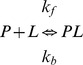
(12)


Assuming that the forward reaction is diffusion-limited, the rate of the forward reaction (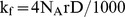) was found to be 4×10^9^ M^−1^s^−1^. *D* = 1×10^−5^ cm^2^s^−1^ is the diffusion coefficient of the ligand [39], *r* (0.4 nm) and N_A_ are typical van der Waals radius and Avogadro’s number, respectively. By using association constant of BBE-BSA complex (K_a_ = k_f_/k_b_ = 6×10^4^) and k_f_ as obtained above, the residence time of the complex (τ ) was found to ∼13 µs at 298 K, which is similar to our earlier report on interaction of curcumin with α-synuclein [16].

### Location of BBE Binding Site on BSA Subdomain IIIA

It is generally accepted that the three domains of serum albumin unfold and refold sequentially. It has been established that domain III unfolds first followed by domain I and the domain II. Urea induced unfolding of BSA and HSA followed by domain specific ligand binding studies showed that domain III unfolded completely between 1 and 3 M urea concentrations without affecting the native conformation of domain I and II [40], [41]. In this study we used this information to locate the binding site of BBE [18].

**Figure 5:**
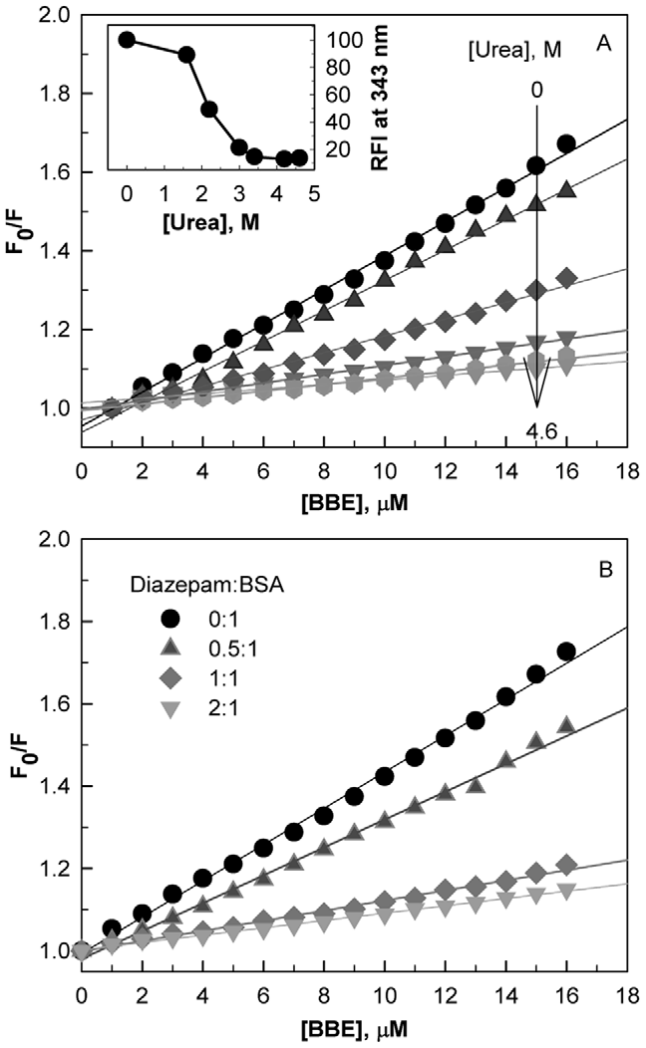
The location of the BBE binding site using unfolding mechanism of BSA and site specific marker. Stern-Volmer plots of BBE-induced fluorescence quenching of BSA with increasing concentration of urea (A). The inset shows the effect of increasing concentrations of urea on the BBE-induced fluorescence quenching of the protein at a fixed BBE/BSA molar ratio of 2∶1. Site specific marker displacement experiments.Stern-Volmer plots of BBE-induced fluorescence quenching of BSA pre-incubated with different concentrations of diazepam, a subdomain IIIA site marker (B).

Stern-Volmer plots of BBE induced quenching of BSA fluorescence in the absence and presence of different concentrations of urea are shown in Figure 5A. As can be seen, a continuous decrease in the K_sv_ is observed between 1 and 3.0 M urea concentrations (Figure 5A and inset Figure 5A), which suggests a continuous loss of BBE-BSA complex formation with increasing urea concentrations. The complete unfolding of domain III [40], [41] and complete loss of BBE binding at 3.0 M urea concentration indicate that the BBE binding site might be located on domain III of the protein.

In order to confirm the BBE binding domain and assign an exact binding location on BSA subdomain, we used diazepam as a site marker of domain IIIA [25]. Figure 5B shows the results of site marker competitive displacement experiments in the form of Stern-Volmer plots. We find that the BBE-induced K_sv_ of BSA decreases continuously as the diazepam/BSA molar ratios increase and no BBE binding was observed at diazepam/BSA molar ratios of 1∶1 and above. This suggests that BBE and diazepam share the same binding site in the subdomain IIIA of BSA and BBE (K_a_ ∼6×10^4^) binds with lower affinity than diazepam (K_a_ ∼10^6^ M^−1^) [42].

### The Effect of BBE Binding on the Conformation of BSA

#### Fluorescence studies

**Figure 6:**
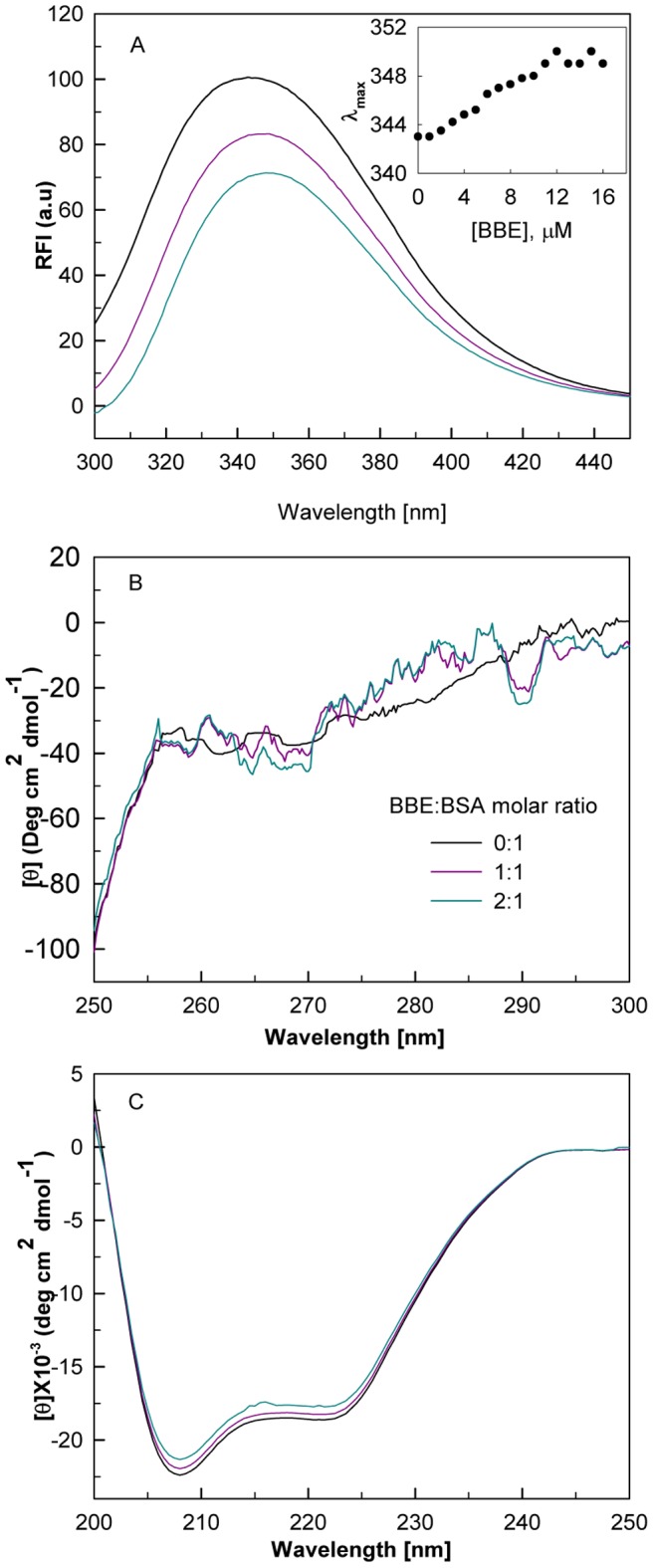
The BBE-induced conformational alterations in BSA. The intrinsic fluorescence (A), near-UV CD (B) and far-UV CD (C) spectra of BSA in the presence of BBE/BSA molar ratios of 0∶1, 1∶1 and 2∶1, respectively. The inset of figure A shows the changes in emission maximum (λ_max_) with increasing BBE concentrations.

The intrinsic fluorescence properties such as intensity and emission maxima (λ_max)_ have been used to monitor the extent of solvation of protein core and as a probe for tertiary structure. Figure 6A shows the effect of BBE binding on the intrinsic fluorescence of BSA in the presence of BBE/BSA molar ratios of 0∶1, 1∶1 and 2∶1. The fluorescence spectra of the BBE-BSA complex show distinct quenching with red shifts of ∼6 nm at highest concentrations of BBE studied. As can be seen from the inset of Figure 6A, BBE induced red shifts are gradual and concentration dependent. These data indicate that BBE binding induces conformational changes in BSA, which bring Trp134 or Trp212 to a more hydrophilic environment.

#### Near-UV circular dichroism studies

To investigate the changes in the tertiary structure of the protein in details, CD measurements in the near-UV region (250–300 nm) were performed in the absence and presence of different BBE/BSA molar ratios. Figure 6B shows the near-UV CD spectra of BSA in the absence and presence of 1∶1 and 2∶1 BBE/BSA molar ratios. In the absence of the ligand two visible minima around 262 and 268 nm are observed, which are similar to the previous reports [43]. The ellipticity 268/262 ratios of the protein in the absence and presence of the ligand are observed to be 0.93 and 1.16, 1.31, respectively (Table 2). On addition of BBE, the increase in the ellipticity ratio is observed which is due to the appearance of a more prominent minimum at 268 nm and a maximum at 262 nm. These changes in the shape of near-UV CD spectrum in the presence of ligand indicate alteration in the tertiary structure of BSA upon binding to the drug.

#### Far-UV circular dichroism studies

The changes in the secondary structure contents of BSA on BBE binding were monitored by far-UV CD measurements between 200 and 250 nm Figure 6C shows the far-UV CD spectra of BSA in the absence and presence of BBE/BSA molar ratios of 1∶1 and 2∶1. In the absence of the ligand, BSA shows two negative minima at 208 and 222 nm, characteristic features of helical proteins. The decrease in the magnitude of mean residue ellipticities [θ] at 222 and 208 nm are indicative of loss of helical structures of BSA in the presence of the ligand. The secondary structural contents (α-helices and β-strands) as calculated by analysis of the CD spectra using K2D3 deconvolution software [21] are shown in Table 2. The values of secondary structures obtained for free BSA are in good agreement with the values reported in the literature [44]–[46]. Analysis of the CD spectra using K2D3 deconvolution software [21] shows a decrease in the alpha-helical contents of BSA without inducing any other regular secondary structure (β-strand) (Table 2).

### The effect of BBE on Unfolding Behavior and Stability of BSA

**Figure 7:**
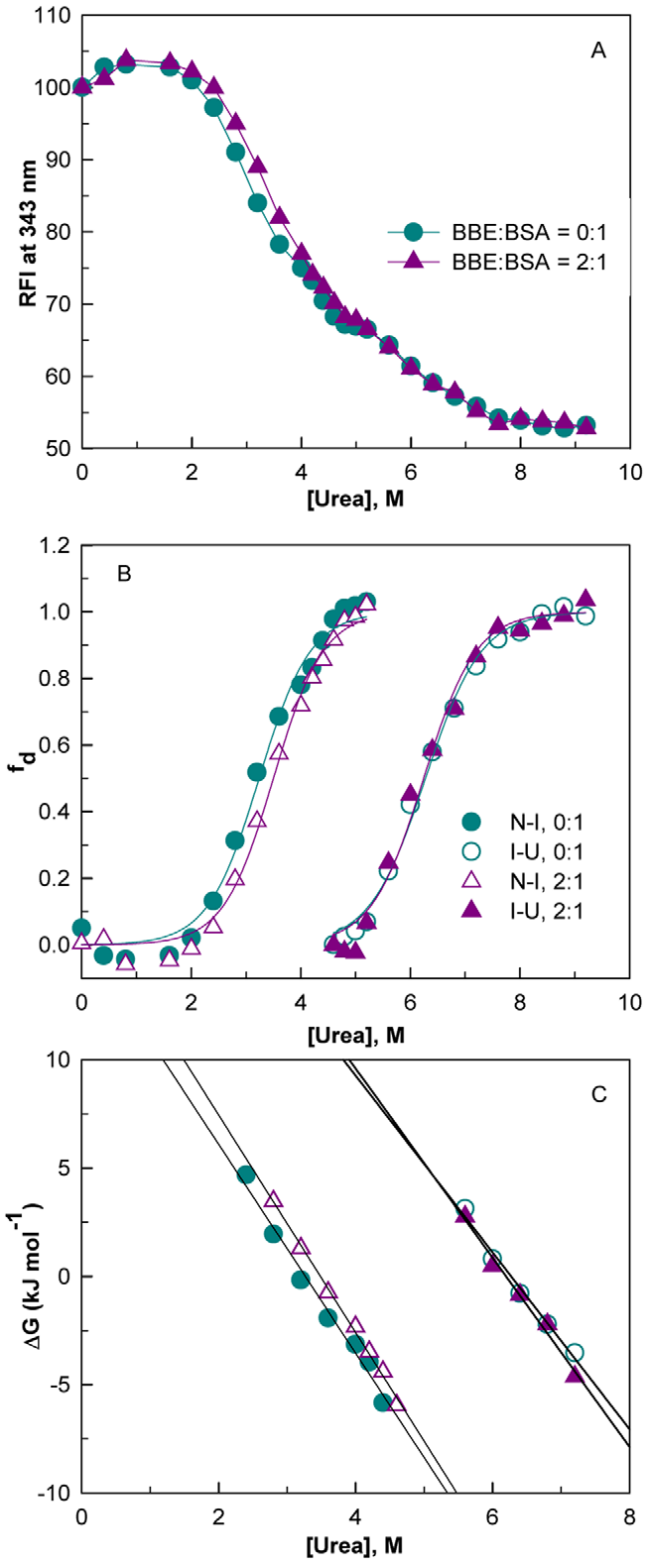
The equilibrium unfolding process and stability of BBE-BSA complex. The urea-induced unfolding profile of BSA and BBE-BSA complex at BBE/BSA molar ratios of 0∶1 and 2∶1 monitored by intrinsic fluorescence of the protein (A). The fraction denatured (f_d_) versus [urea] plots for N
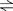
I and I
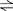
U transitions of the protein and complex. Lines represent the nonlinear regression fitting of the data according to equation 7 (B). The dependence of free energy change as a function of urea concentrations for the transitions shown in Figure B (C).

The effect of a molecule on the unfolding behavior of serum albumin is crucial for the molecule to be qualified as a drug. The effects of BBE binding on the equilibrium unfolding of BSA was investigated by urea-induced denaturation as monitored by measurements of fluorescence intensity at 343 nm after exciting the protein at 280 nm. Figure 7A shows the urea-induced denaturation transitions of BSA in the absence and presence of BBE/BSA molar ratio of 2∶1. As reported earlier [40] in the absence of the ligand, unfolding starts ∼2.4 M urea and follows a two-step, three state transition with the accumulation of an equilibrium intermediate state (I) around 4.8–5.2 M urea concentrations. The addition of the ligand at a BBE/BSA molar ratio of 2∶1 shifts the native-to-intermediate state (N
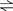
I) transition towards higher urea concentrations without significantly affecting the intermediate- to-unfolded state (I
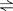
U) transition (Figure 7A and 7B). The free energy of stabilization (ΔΔG^H^_2_^O^) in the presence of BBE was found to be 2.06±0.29 and 1.99±0.27 kJ mol^−1^ as determined by linear and non-linear fittings of intrinsic fluorescence data, respectively (Figure 7B and 7C). The stabilization occurrs in the first transition, which corresponds to the formation of intermediate state (I), whereas the second transition, corresponding to the unfolding of I state to U state remains unaffected (Table 3). Since N to I state transition is characterized by unfolding of domain III, stabilization of the N 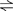 I transition also indicates the binding of the ligand in domain III of BSA [40], [41].

**Table 3:** The effect of 1,2-*Bis*(2-Benzimidazolyl) 1,2-Ethanediol binding on unfolding transition of BSA.

BBE/BSA Molar ratio	Regression	ΔG_N 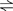 I_ (kJ mol^−1^)	ΔG_I 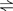 U_(kJ mol^−1^)	ΔG^H2O^ (kJ mol^−1^)	kJ mol^−1^ (ΔΔG^H2O^)
0∶1	Nonlinear	17.19±1.39	15.23±1.45	32.42±2.84	
	Linear	15.72±1.08	9.99±1.23	25.71±2.31	
2∶1	Nonlinear	19.09±1.24	15.32±1.87	34.41±3.11	1.99±0.27
	Linear	17.47±1.71	10.30±1.89	27.77±3.60	2.06±0.29

### Computational Study on the Binding of BBE with Subdomain IIIA of BSA

**Figure 8:**
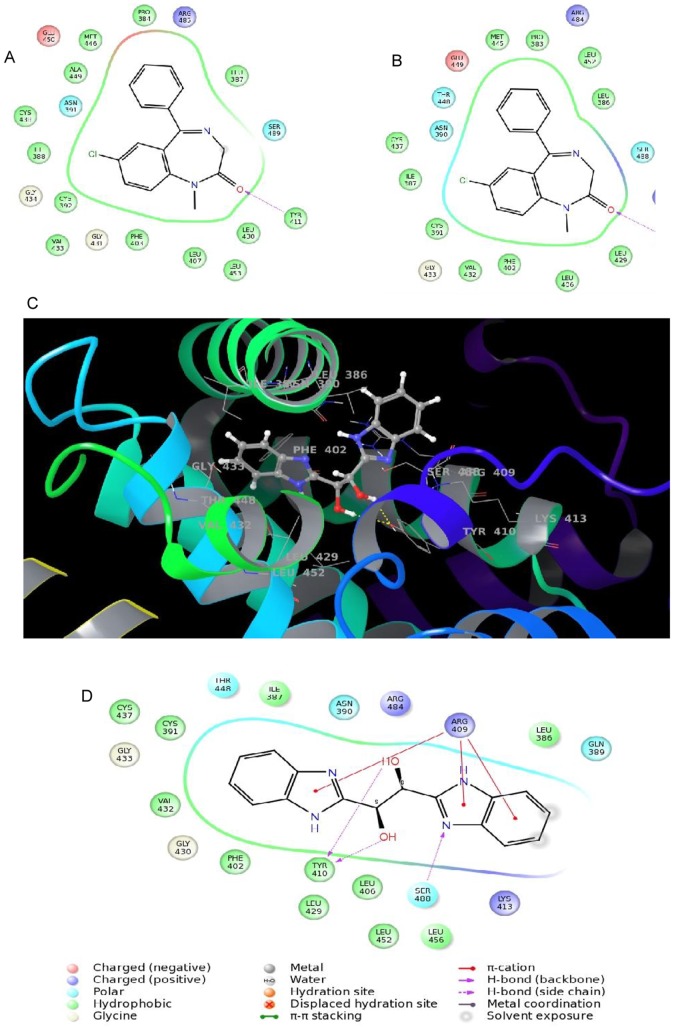
Binding analysis of BBE in the subdomain IIIA of BSA by molecular modeling. 2D ligand interaction diagrams for diazepam with the subdomain IIIA of HSA (A) and BSA (B). 3D interaction diagram for BBE with the subdomain IIIA of BSA (C). 2D BBE interaction diagram with subdomain IIIA of BSA.

Our experimental studies showed that BBE bound to the diazepam binding site on sub domain IIIA of BSA. Here we further study it by a computational method to strengthen our findings. Since no ligand is bound to the X-ray crystal structure of BSA (PDB code 3V03), the interaction site was determined based on the X-ray crystal structure of HSA bound to diazepam (PDB code 2BXF) [25], [26]. The two proteins HSA and BSA have high sequence similarity with 100% match in the subdomain IIIA. The two proteins were structurally aligned to a single coordinate frame, subsequent to which the atom coordinates of diazepam were extracted from HSA into the coordinate frame of BSA. The 10 Å^3^ grid was built with diazepam at the grid center for the docking. The protocol was validated by the efficient docking of diazepam in subdomains IIIA pocket of HSA and BSA having less than 1.0 Å root mean square deviation (RMSD) of the docking poses to that of the bound structure. Identical interactions were observed for diazepam with the amino acids in the IIIA subdomain of HSA and BSA, especially the hydrogen bonding with Tyr411 and Tyr 410 with the respective proteins (Figure 8a and 8b).

### Binding Analysis of BBE in the Subdomain IIIA of BSA

The ligand shows a snug fit in between the three helices of the subdomain IIIA of BSA as depicted in Figure 8C. Being a symmetric molecule and based on the pocket made by the three helices, a single orientation was identified for BBE in the cavity. The phenyl ring of one benzimidazole was surrounded by the hydrophobic residues namely, Ile387, Cys391, Phe402, Val432 and Cys437 while the other phenyl on the second benzimidazole was exposed to solvent. Hydrogen bond interactions were seen between hydroxyl groups of ethane-1, 2-diol fragment with Tyr410 and benzimidazole sp^2^ nitrogen atom with Ser488. The 2D ligand interaction diagram (Figure 8D) also revealed π-cation interactions of the imidazole rings of benzimidazole with amino acid Arg409.

The association constant (K_a_) obtained from modeling (10^4^ M^−1^) is very much consistent with the K_a_ value (10^4^ M^−1^) calculated from fluorescence quenching data (Table 1, Table 4). Moreover, the GlideScore XP for BBE shows a lower affinity (25.01 kJ mol^−1^) to BSA than the score computed for diazepam (34.51 kJ mol^−1^) (Table 4). These docking scores support the experimental ligand binding and displacement studies wherein BBE was found inept to displace diazepam from the subdomain IIIA.

**Table 4:** Docking score (GlideScore XP) as calculated for the best poses for diazepam and BBE.

Protein	Ligand	GlideScore (kJ mol^−1^
HSA (subdomain IIIA)	Diazepam	−33.77
BSA (subdomain IIIA)	Diazepam	−34.51
BSA (subdomain IIIA)	BBE	−25.04

## Discussion

Serum albumin is the main carrier of drugs in the mammalian circulatory system including for humans. It contains many binding sites for a large variety of substances, two of which, Sudlow site I located in domain IIA and Sudlow site II located in domain IIIA are the main sites for drug binding [11], [12]. The drug-serum albumin binding parameters and drug induced conformational changes determine the distribution, efficacy, clearance and brain penetration of the drug [11], [14], [15]. Therefore, the drug-protein interaction study is an important part of the drug design.

In this study, we have investigated the binding parameters, thermodynamic parameters, structural changes and conformational stability associated with the BBE interaction with bovine serum albumin (BSA). The binding data with urea-unfolded BSA and in the presence of a site-specific marker suggest one binding site for the molecule, which is located in subdomain IIIA of BSA. On the basis of molecular modeling, large negative values of ΔH, ΔG and small positive values of ΔS for BBE-BSA complex formation, we proposed that BBE readily bound BSA mainly through hydrophilic interaction such as hydrogen bonding and hydrophobic interactions in subdomain IIIA. The computational studies indicated hydrogen bonding between hydroxyl groups of BBE with Tyr410 and benzimidazole nitrogen atom with Ser488 and hydrophobic interaction between phenyl ring of one benzimidazole of the ligand and hydrophobic residues namely, Ile387, Cys391, Phe402, Val432 and Cys437.

Intrinsic fluorescence, near-UV and far-UV CD data suggest that the interaction of BBE with BSA causes small but significant alterations in both tertiary and secondary structure of the protein. The study of small molecules-induced conformational changes is crucial for structure-based rational drug design. Small molecules binding to serum albumin are known to cause physiologically relevant and as well as harmful conformational changes. For example, the binding of long chain fatty acid to serum albumin cause alteration in the microenvironments of the lone free sulfhydryl group (Cys-34) and regulate the radical-trapping antioxidant activity [47]. Some small-molecule-induced structural changes modulate the β-lactamase activity of the albumin and may increase the drug resistance through a non-microbial process [48]. The binding of small molecules to serum albumin can also alter the conformation of binding sites for other drugs and thus modulate the ADMET (absorption, distribution, metabolism, excretion and toxicity), which is a major challenge in drug design.

Urea-induced unfolding of BSA-BBE complex indicated that the native state is stabilized by ∼2kJ mol^−1^ relative to intermediate state and the I
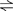
U transition is unaffected. Binding to the native state is generally known to increase the stability of proteins [49]. Therefore, these results suggested that the molecule bound to the native conformation of domain III. These findings and conformational alterations indicate that binding of the BBE to domain IIIA may be inducing conformational changes in domain I and/or domain II, which brought the tryptophan residues to a more polar environment. Similar ligand-induced propagation of conformational changes between domains has also been reported in other protein [50].

This work yields information about the mechanism of interaction of synthetic multi-microbial inhibitor, 1,2-bis(2-benzimidazolyl)-1,2-ethanediol (BBE) with bovine serum albumin. We observed that its binding mechanism is similar to the drugs, which bind to domain IIIA of the serum albumin. The BBE molecule, which is a tridentate ligand, can be prepared by simple Phillips reaction and offers many sites for derivatization. Therefore, this molecule or its derivatives could be a good drug candidate for the treatment of multimicrobial infections of animals and/or human.
